# Relationship between histological findings of vastus lateralis muscle and function after total hip arthroplasty in patients with hip fracture: a prospective cohort study

**DOI:** 10.2340/17453674.2024.42099

**Published:** 2024-10-28

**Authors:** Suk-Kyoon SONG, Ji-Hyun HWANG, Jin-Woo BAE, Hoon-Kyu OH, Myung-Rae CHO

**Affiliations:** 1 Department of Orthopaedic Surgery, Daegu Catholic University Medical Center; 2 College of Medicine, Daegu Catholic University; 3 Department of Pathology, Daegu Catholic University Medical Center, South Korea

## Abstract

**Background and purpose:**

We aimed to examine the histological characteristics of vastus lateralis muscles in patients undergoing total hip arthroplasty (THA) following femoral neck fractures and to explore the correlation between muscle fiber types and postoperative functional recovery.

**Methods:**

34 patients undergoing THA for femoral neck fractures were included. A biopsy of the vastus lateralis muscle was performed during surgery, followed by immunohistochemical staining. Subsequently, image analysis was conducted to measure the average area of muscle fiber types and the number of type I and II muscle fibers, and the ratio of the area and the number of type II muscle fibers. Functional recovery was assessed 2 weeks post-surgery using the Short Physical Performance Battery (SPPB).

**Results:**

A significant positive correlation was observed between type II muscle fibers and SPPB scores. The ratio of type II muscle fiber area and number strongly correlated with the SPPB scores, indicating a robust static association. The average area of type II fibers showed a strong correlation (r = 0.63, P < 0.001), as did the number of type II fibers (r = 0.53, P = 0.001). Moreover, the ratio of type II muscle fiber area and number significantly correlated with SPPB scores (area: r = 0.77, P < 0.001; number: r = 0.51, P = 0.002), indicating that larger and more numerous type II fibers are associated with better physical performance.

**Conclusion:**

The reduction of type II muscle fibers was strongly correlated with a low SPPB postoperative functional recovery in patients who underwent THA following femoral neck fractures.

Sarcopenia, characterized by reduced muscle mass and strength, impairs functionality, particularly as aging progresses [1]. With age, a decline in muscle mass, strength, and reaction time increases fall risk, contributing to severe outcomes such as hip fractures with high mortality rates [2]. Recent studies have identified sarcopenia as a significant risk factor for hip fractures, with poorer postoperative recovery and higher mortality compared with patients without sarcopenia [3-6]. Given the high morbidity and mortality associated with hip fractures, proactive prevention and treatment strategies for sarcopenia are essential.

Aging leads to a 6% decline in the skeletal muscle every decade after middle age [7]. Skeletal muscle consists of type I and II fibers, which coexist and determine muscle function. In age-related sarcopenia, muscle wasting is primarily caused by a reduction in type II muscle fibers [8-10]. Consequently, the loss of type II fibers, responsible for rapid force generation, quick response, and power, increases fall risk [11]. Recent changes in sarcopenia diagnostic criteria emphasize physical activity [12], suggesting that understanding the correlation between histological observations and patient function could provide crucial data for precise diagnosis and postoperative functional assessment in patients with hip fracture. This reflects the understanding that histological observations are also crucial for assessing the muscle’s functional and qualitative state. However, the impact of changes in type II muscle mass on postoperative functional recovery in these patients remains inconclusive. Therefore, this study aimed to explore the number and area of type I and II muscle fibers in patients with femoral neck fractures and investigate their correlation with postoperative functional recovery measured with the Short Physical Performance Battery (SPPB) following total hip arthroplasty (THA).

## Methods

### Study design and participants

From June 2022 to June 2023, patients with femoral neck fractures who previously had full ambulatory capacity without assistive devices, indicated by a Koval walking ability score of 1 [13], were enrolled in this prospective, observational cohort study. Patients with a history of ipsilateral lower limb surgery or neuromuscular disorders were excluded. Upon admission, a structured questionnaire was administered to assess preoperative hip function using the Harris Hip Score and body mass index (BMI). Additionally, all participants underwent dual-energy X-ray absorptiometry scans to measure bone density and muscle mass, establishing baseline health metrics for subsequent analyses.

The study is reported according to STROBE guidelines.

### Surgical procedure and muscle biopsies

All surgical procedures were performed using a modified Hardinge approach with patients positioned laterally. A 12 cm skin incision, starting 5 cm proximal to the greater trochanter tip and traversing through the center of the greater trochanter tip, was extended distally. The fascia lata was split, and dissection proceeded from the anterior one-third point of the gluteus medius to the midpoint of the vastus lateralis. During this process, muscle specimens of the vastus lateralis (5 mm × 5 mm × 5 mm) were harvested perpendicularly to the muscle fibers. Following joint capsulotomy, the fracture site was exposed, and the head fragment was removed following femoral neck cutting. Subsequently, the acetabulum was reamed and a cup was inserted considering anteversion and inclination. Porous-coated cementless acetabular cups (Trilogy, Zimmer, Warsaw, IN, USA) and highly cross-linked polyethylene acetabular liners (Longevity, Zimmer, Warsaw, IN, USA) were utilized for all cases. The femoral canal was prepared, and the femoral stem (M/L taper; Zimmer, Warsaw, IN, USA) was inserted using standard press-fit techniques, ensuring longitudinal and rotational stability. The head assembly was securely attached. Patients were encouraged to sit on the first postoperative day and stand with support as able.

### Immunohistochemistry

Immunohistochemistry procedures were performed on 5 μm paraffin-embedded tissue sections using the Bond Polymer Intense Detection System (Leica Microsystems, Mount Waverley, VIC, Australia) per the manufacturer’s instruction with minor modifications. The sections were then deparaffinized with Bond Dewax Solution (Leica Microsystems, Mount Waverley, VIC, Australia), followed by antigen retrieval using Bond ER Solution (Leica Microsystems, Mount Waverley, VIC, Australia) for 30 min at 100℃. Endogenous peroxidase activity was quenched by incubation in hydrogen peroxide for 5 min. Moreover, sections were incubated for 15 min at ambient temperature with Recombinant Anti-Fast Myosin Skeletal Heavy chain + MYH4 antibody (EPR22880-64) and Recombinant Anti-Slow Skeletal Myosin Heavy chain antibody (EPR22697-17).

### Measurement of the areas and quantities of respective muscle fiber

**Figure 1:**
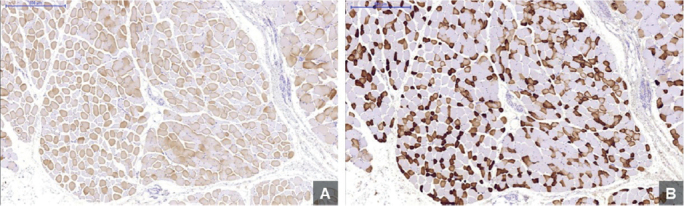
Optical microscope images of muscle fibers at 5x magnification. A shows Type I muscle fibers. B represents Type II muscle fibers. Images were processed with panoramic viewer software.

The stained sections were scanned using a Panoramic MIDI (3DHistech, Budapest, Hungary), and the data was uploaded using imaging software (panoramic viewer) (Figure 1).

**Figure 2:**
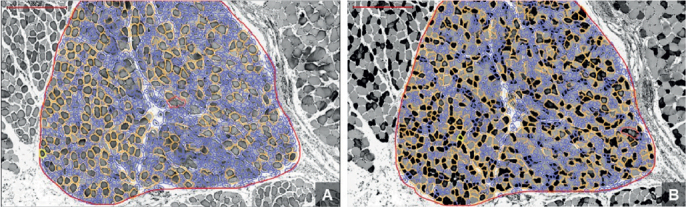
Automated differentiation and quantification of muscle fibers using QuPath software. A displays the region of interest with Type I muscle fibers outlined, while B shows Type II muscle fibers within the same region of interest. The analysis includes both area measurements and fiber counts.

Efforts were made to cut tissue sections perpendicularly to minimize errors. In case of oblique cutting, clusters with the smallest average muscle fiber diameter were selected for measurement. Using QuPath software (https://qupath.github.io/), type I and II muscle fibers were automatically distinguished within the region of interest, and their respective areas and quantities were measured (Figure 2).

QuPath’s feature-based algorithm differentiates muscle fiber types by analyzing morphological characteristics, such as color, size, and shape. This method is particularly useful for analyzing color variations of stained fibers, serving as a critical criterion for the automated classification of muscle fibers. This approach, based on the diameter of smaller fibers, is particularly effective in minimizing measurement errors in cross-sectioned fibers. Our analysis leveraged cell analysis and positive cell detection, with the setup parameters listed in Table 1. A single-threshold option was used owing to the grayscale configuration of the images, allowing for more precise detection and categorization.

**Table 1:** Setup parameters for cell analysis and positive cell detection

Setup parameters/Detection image	Optical density sum
Intensity parameters	
Threshold	0.1
Max background intensity	2
Split by shape	Yes
Exclude DAB (membrane staining)	No
General parameters	
Smooth boundaries	No
Make measurements	Yes
Intensity threshold parameters	
Score compartments	Cell: DAB OD mean
Single threshold	Yes

DAB = 3,3’-diaminobenzidine; OD = optical density

### Short Physical Performance Battery as a measure of physical performance

All participants underwent an SPPB assessment 2 weeks postoperatively, immediately before discharge. The SPPB test was designed to measure functional status and physical performance [14]. It includes 3 objective tests of lower body function: (i) a timed 4-m walk at a normal pace, (ii) 5 timed, repeated chair stands measuring the time required to perform 5 rises from a chair to an upright position as fast as possible with the arms kept across the chest, and (iii) 3 standing balance tests (side-by-side, semi-tandem, and tandem stands), with the maximum score awarded for successfully standing for 10 s in each test. Each category had a maximum score of 4, with a total possible score of 0–12, where higher scores indicated better lower body function. The sum of the 3 individual test items equaled the SPPB summary score.

### Statistics

Continuous variables were expressed as mean (SD), and categorical variables as absolute frequencies and percentages. Pearson’s correlation analysis was used to determine correlations between SPPB scores and variables, such as age, sex, activity level, BMI), bone mineral density (BMD), and skeletal muscle fiber parameters (fiber area and number). Univariate and multiple linear regression analyses were performed to detect potential independent predictors of functional recovery (SPPB score). Regression analysis assumptions—normality, independence, linearity, and equal variance—were confirmed. The variance inflation factor (VIF) value was used to diagnose multicollinearity; variables (VIF > 10) were excluded from the multivariate analysis. Multivariate analysis was adjusted for age, sex, and BMI.

Correlation coefficients (r) were used to measure the strength of the relationship between muscle fiber characteristics and SPPB scores. We adopted widely accepted thresholds to interpret these coefficients, as suggested by Cohen: small (~0.10), moderate (~0.30), and strong (≥0.50) [15]. P ≤ 0.05 was considered statistically significant. All analyses were performed using SPSS version 25.0 (IBM Corpn, Armonk, NY, USA). The sample size was determined based on a prospective study evaluating the relationship between sarcopenia and histological findings in the vastus lateralis muscle [10]. 30 patients were required to achieve 80% power at alpha = 0.05.

### Ethics, use of AI, funding, and disclosure

The study protocol was reviewed and approved by the Institutional Review Board of Daegu Catholic University Medical Center (approval number: CR-21-074), and all participants provided written informed consent. While drafting the manuscript, we used ChatGPT-4 for assistance with English-language composition. However, all content was carefully reviewed and revised by the authors to ensure accuracy and quality. The authors disclose receipt of the following financial support for the research, authorship, and/or publication of this article: this work was supported by the National Research Foundation of Korea (NRF) grant funded by the Korea government (MSIT) (No. RS-2022-00165702). Complete disclosure of interest forms according to ICMJE are available on the article page, doi: 10.2340/17453674.2024.42099

## Results

**Figure 3:**
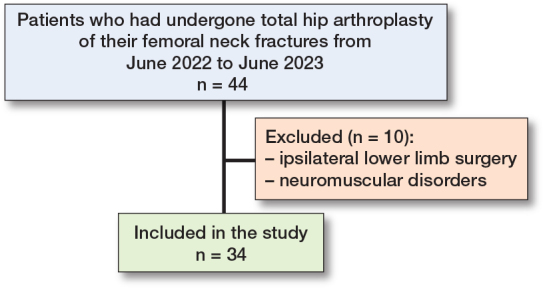
Flowchart of patients.

Figure 3 provides a flowchart of the included patients. Table 2 presents the general characteristics of the study participants. The participants’ ages ranged from 60–88 years, with a mean age of 70.5 years. The cohort included 11 males (32%) and 23 females (68%), with an average BMI of 24.0. The overall mean SPPB score for the cohort was 6.1. When analyzed by sex, males had a mean SPPB score of 5.6, while females had a mean SPPB score of 6.3, showing functional performance between the 2 groups.

**Table 2:** General characteristics of study participants (N = 34). Values are mean (standard deviation) unless otherwise specified

Variable	Value
Age, years	70.5 (7.7)
Body mass index	24.0 (3.6)
Sex (female/male), n	23/11
mHHS, points	59.3 (14.6)
Hip BMD, T-score	–2.2 (1.0)
Spine BMD, T-score	–1.9 (1.2)
Type I muscle fiber av erage area, μm²	643 (104)
Type II muscle fiber average area, μm²	476 (83)
Ratio of type II muscle fiber area	0.4 (0.1)
Type I fiber number	25.4 (8.9)
Type II fiber number	24.5 (4.9)
Muscle fiber number ratio (Type II/Type I)	1.1 (0.5)
SPPB score	6.1 (2.4)

mHHS = modified Harris Hip Score; BMD = bone mineral density; SPPB = Short Physical Performance Battery.

**Figure 4:**
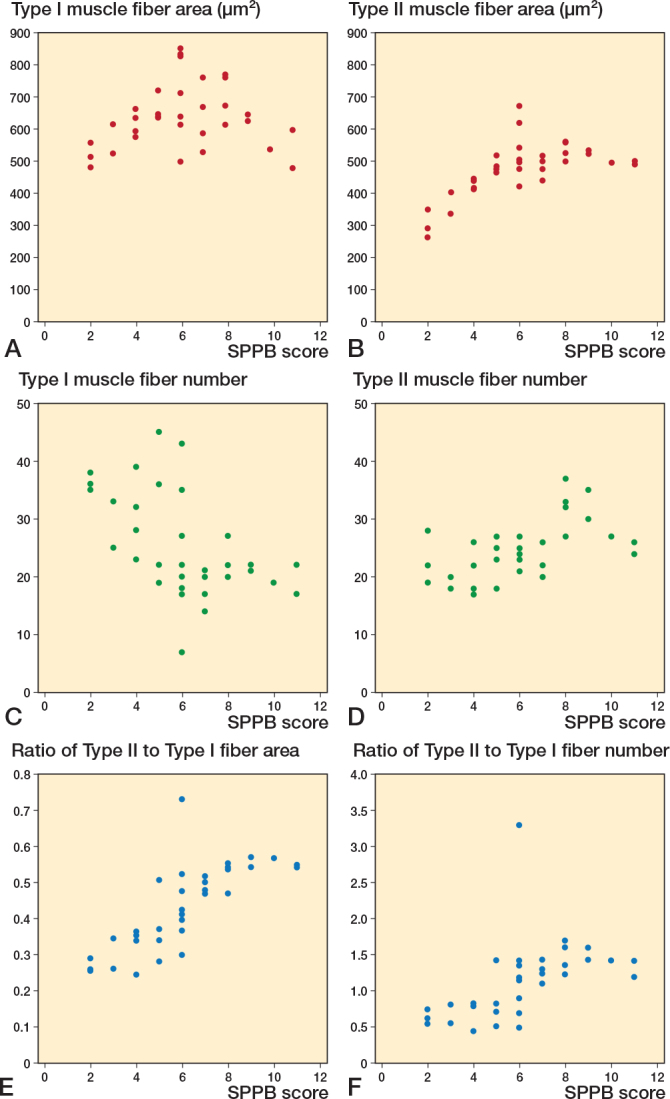
Scatter plots between vastus lateralis muscle fibers and Short Physical Performance Battery (SPPB) scores. Correlation between (A) Type I area, (B) Type II area, (C) Type I fiber number, (D) Type II fiber number, (E) ratio of Type II to Type I fiber area, (F) ratio of Type II to Type I fiber number and SPPB score.

Our analysis examined the relationship between muscle fiber characteristics and SPPB scores (Figure 4). Type 1 fiber count showed a negative correlation with SPPB scores, while the average area, number, percentage of area, and the number of type II fibers correlated positively with SPPB scores, as indicated in Table 3.

**Table 3:** Histological analysis of vastus lateralis muscle fibers and their partial correlation with SPPB score

Variable	SPPB score	
	Correlation coefficient (r)	P value
Type I muscle fibers average area	0.12	0.5
Type II muscle fibers average area	0.63	< 0.001
Type I muscle fibers number	–0.54	0.001
Type II muscle fibers number	0.53	0.001
Ratio of type II muscle fibers area	0.77	< 0.001
Ratio of type II muscle fibers number	0.51	< 0.001

SPPB = Short Physical Performance Battery.

Type II muscle fibers demonstrated a positive correlation with SPPB scores regarding average fiber area (r = 0.63, P < 0.001). Type II muscle fiber numbers also positively correlated with SPPB scores (r = 0.53, P = 0.001), whereas type I fiber numbers indicated a significant negative correlation with SPPB scores (r = –0.54, P = 0.001). However, the average areas of type 1 fibers did not significantly correlate with SPPB scores.

Regarding the percentage of area and number, type II muscle fibers were significantly correlated with the SPPB score (area: r = 0.77, P < 0.001; number: r = 0.51, P = 0.002).

**Figure 5:**
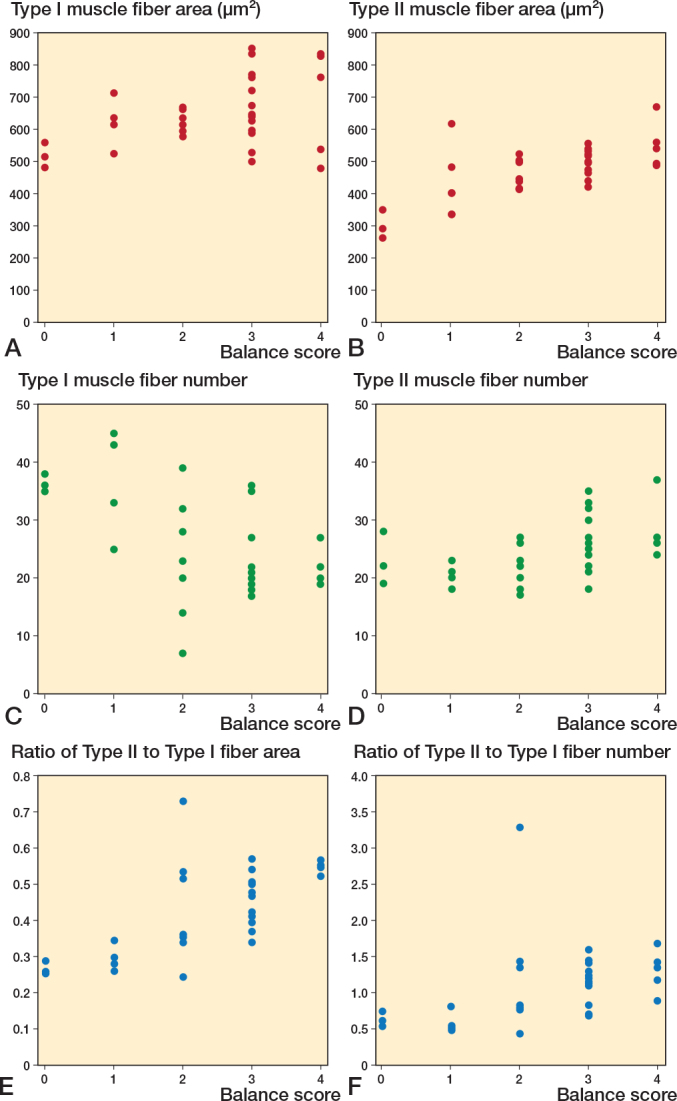
Scatter plots between vastus lateralis muscle fibers and balance score. Correlation between (A) Type I area, (B) Type II area, (C) Type I fiber number, (D) Type II fiber number, (E) ratio of Type II to Type I fiber area, (F) ratio of Type II to Type I fiber number and balance score.

**Figure 6:**
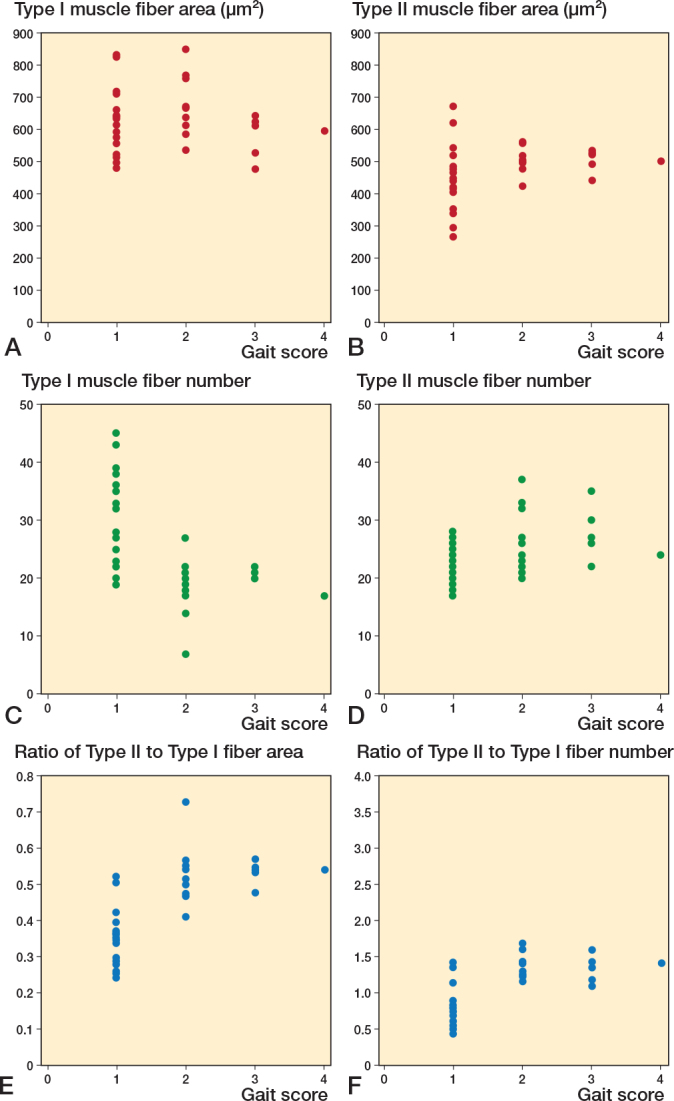
Scatter plots between vastus lateralis muscle fibers and gait scores. Correlation between (A) Type I area, (B) Type II area, (C) Type I fiber number, (D) Type II fiber number, (E) ratio of Type II to Type I fiber area, (F) ratio of Type II to Type I fiber number and gait score.

**Figure 7:**
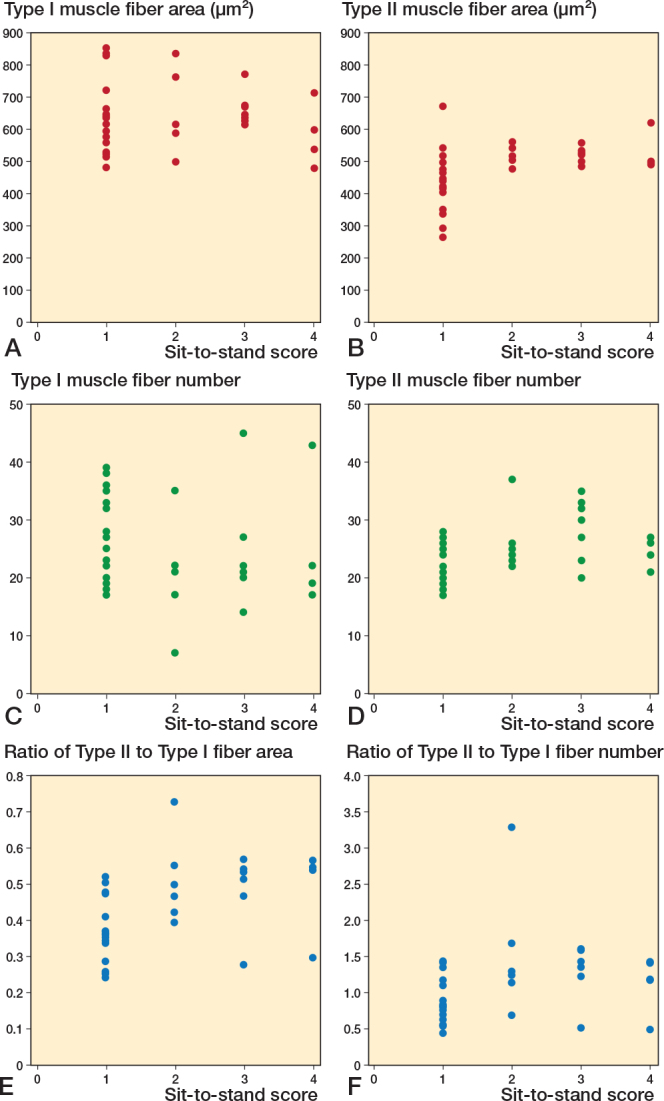
Scatter plots between vastus lateralis muscle fibers and sit-to-stand scores. Correlation between (A) Type I area, (B) Type II area, (C) Type I fiber number, (D) Type II fiber number, (E) ratio of Type II to Type I fiber area, (F) ratio of Type II to Type I fiber number and sit-to-stand score.

Further analysis of SPPB score components—balance, gait speed, and sit-to-stand scores—revealed significant correlations between these components and specific features of the vastus lateralis muscle fibers. As illustrated in Figure 5, we focused on the average area and number of both type I and II muscle fibers, and their respective ratios, which showed significant correlations with the balance score. The correlation between these muscle fiber characteristics and gait speed demonstrated significant associations (Figure 6). Notably, the number of type I and II muscle fibers, with the ratios of type II muscle fiber area and number, correlated with gait speed. Moreover, analysis of sit-to-stand scores revealed significant correlations (Figure 7), with the average area, number, and area ratios of type II muscle fibers strongly associated with sit-to-stand performance. Age was negatively correlated with SPPB scores (r = –0.36, P = 0.04).

Univariate regression analysis identified age, hip BMD, and skeletal muscle fiber parameters as predictors of SPPB scores 2 weeks post-surgery. However, adjusted multivariate regression analysis demonstrated that the percentage and quantity of type II fibers were independent predictors of better functional recovery across all age groups 2 weeks after THA (Table 4).

**Table 4:** Univariate and multivariate analysis for variables significantly associated with SPPB scores 2 weeks post-surgery

Variable	B (CI)	P values
Univariate analysis		
Age, years	–0.11 (–0.22 to –0.007)	0.04
Body mass index	–0.17 (–0.40 to 0.066)	0.2
Sex (female/male)	0.63 (–1.2 to 2.4)	0.5
modified Harris Hip Score	0.014 (–0.045 to 0.073)	0.6
Skeletal muscle index	0.32 (–0.58 to 1.2)	0.5
Spine bone mineral density	0.41 (–0.33 to 1.1)	0.3
Hip bone mineral density	0.90 (0.086 to 1.7)	0.03
Muscle fibers average area		
type 1	0.003 (–0.005 to 0.011)	0.5
type 2	0.018 (0.010 to 0.026)	< 0.001
Muscle fibers number		
type 1	–0.15 (–0.23 to –0.063)	0.001
type 2	0.26 (0.11 to –0.41)	0.001
Ratio of type 2/1 muscle fiber		
area	16 (11 to 20)	< 0.001
number	2.3 (0.90 to 3.7)	0.002
Multivariate analysis		
Age, years	–0.03 (–0.10 to 0.04)	0.4
Hip bone mineral density	0.14 (–0.45 to 0.72)	0.6
Type 2 muscle fibers		
average area	0.003 (–0.003 to 0.010)	0.3
Muscle fibers number		
type 1	0.024 (–0.096 to 0.14)	0.7
type 2	0.048 (–0.089 to 0.19)	0.5
Ratio of type 2/1 muscle fiber		
area	29 (15 to 44)	< 0.001
number	–3.8 (–5.8 to –1.8)	0.001

SPPB = Short Physical Performance Battery; CI = 95% confidence interval.

## Discussion

We aimed to investigate the correlation between functional recovery and the preservation of type II muscle fibers in patients who underwent THA for femoral neck fractures. We found a significant positive correlation between the quantity and area of type II muscle fibers and SPPB scores, measuring functional recovery at 2 weeks post-surgery indicating that the more type II fibers the better the SPPB scores and function of the patient. Our findings suggest that the area and quantity of type II muscle fibers are involved in postoperative functional recovery.

Geriatric hip fractures are associated with high mortality rates, and recent reports have indicated a profound association between fractures and sarcopenia. Bones and surrounding muscles interact through the secretion of osteokines and myokines via endocrine and paracrine mechanisms, respectively [16,17]. Yoo et al. [18] demonstrated a higher prevalence of sarcopenia in patients with hip fractures than in the general population. Despite the focus on osteoporosis prevention, sarcopenia remains under-addressed. Therefore, identifying a common approach for preventing and treating sarcopenia in future research is essential.

Recent studies have emphasized the importance of type II muscle fibers in maintaining muscle and bone quality [19,20], suggesting that preserving type II muscle fibers in older adults could be a strategic approach to minimizing fall risks and subsequent hip fractures [21]. However, the phenomenon of a more pronounced reduction in type II muscle fibers is particularly significant in patients with age-related sarcopenia [8-10].

According to another study, the diameter of type II muscle fibers in the non-sarcopenia group averages 59.1 μm in men and 42.1 μm in women, while the diameter of type I muscle fibers averages 66.8 μm in men and 57.9 μm in women [10]. These values correspond to areas of approximately 2,743 μm² for type II and 3,505 μm² for type I fibers in males, and 1,392 μm² for type II and 2,633 μm² for type I fibers in females. Conversely, our study found that the area of type II muscle fibers was 476.3 μm² and the area of type I muscle fibers was 643.2 μm², indicating a significant reduction in muscle fiber size in our patient population. This may be explained by muscle atrophy, which is commonly observed in older adults, especially in patients with hip fractures.

In our study, we employed the SPPB to assess physical activity capabilities [14]. Our findings demonstrated a significant positive relationship between the area and quantity of type II muscle fibers and patients’ functional recovery. To the best of our knowledge, no studies have linked histological changes in human skeletal muscle fibers to functional recovery post-hip fracture. These findings indicate the crucial role of type II muscle fibers in patients’ functional recovery.

Type II muscle fibers, known for their rapid response to changes in physical activity and muscle strength, are crucial in preventing falls and facilitating rehabilitation therapy in patients with hip fractures. Many older individuals engage in activities such as walking and stretching, which does not promote type II fiber development. As individuals age, it becomes imperative to actively and consistently participate in strength-training exercises to prevent loss of type II muscle fibers. Furthermore, such exercises can benefit hip BMD increment. Hence, to prevent muscle atrophy and promote functional recovery after proximal femur fractures, it is essential to implement interventions such as exercise, nutrition, and medications to prevent loss and enhance the development of type II muscle fibers in these patients. When discussing interventions preventing the loss and enhancement of type II muscle fiber development, the fibers respond to high-intensity exercise by increasing their size. High-intensity interval training and resistance training can enhance the size and function of type II muscle fibers [22-25]. These training methods not only increase muscle strength and power but also alter muscle fiber composition, thereby increasing type II fiber proportion and contributing to overall body function and health maintenance.

### Limitations

First, this was a single-center study with a small sample size. Second, functional recovery was evaluated only at the 2-week postoperative timepoint. Longer-term follow-up could provide a more comprehensive understanding of the functional recovery process. Third, selected patients may have underlying conditions with unclear diagnoses that could affect skeletal muscle function. Expanding the sample size and conducting multicenter studies would allow for the exploration of independent risk factors affecting functional recovery following hip fracture surgery. Fourth, investigating the skeletal muscle fiber characteristics and the factors influencing type II muscle fiber reduction and their relationships in detail is essential. This would provide new insights into comprehensive interventions for the prevention and enhancement of type II muscle fibers, shaping future research directions.

### Conclusion

A reduction of type II muscle fibers strongly correlated with low SPPB postoperative functional recovery in patients who underwent THA following femoral neck fractures, indicating that type II muscle fibers may have a critical role in postoperative recovery. Preventing their loss and promoting their development is essential for reducing the risk of high-mortality hip fractures and improving recovery outcomes. Hence, rehabilitation exercises and nutritional strategies targeting type II muscle fibers may support this.

## Notes

SKS and MRC developed the study concept and design. MRC and HKO designed and supervised the study. SKS, JHH, and HKO collected data and conducted the study using biopsy samples. SKS, JHH, and JWB performed the statistical analyses and wrote the draft of the manuscript. SKS, MRC, and HKO critically revised the manuscript.

Handling co-editors: Taco Gosens

*Acta* thanks Lene Bergendal Solberg and Hanna C Willems for help with peer review of this manuscript.
